# Metal-based proteasomal deubiquitinase inhibitors as potential anticancer agents

**DOI:** 10.1007/s10555-017-9701-1

**Published:** 2017-10-16

**Authors:** Xin Chen, Qianqian Yang, Lu Xiao, Daolin Tang, Q. Ping Dou, Jinbao Liu

**Affiliations:** 1 Protein Modification and Degradation Lab, School of Basic Medical Sciences, Affiliated Tumor Hospital of Guangzhou Medical University, Guangzhou, China; 2 Department of Surgery, University of Pittsburgh, Pittsburgh, PA 15213 USA; 3 The Molecular Therapeutics Program, Barbara Ann Karmanos Cancer Institute, Detroit, USA; 4 Department of Oncology, Pharmacology and Pathology, School of Medicine, Wayne State University, Detroit, MI 48201-2013 USA

**Keywords:** Proteasome, Deubiquitinase, Metal, DUB inhibitor, Cancer

## Abstract

Deubiquitinases (DUBs) play an important role in protein quality control in eukaryotic cells due to their ability to specifically remove ubiquitin from substrate proteins. Therefore, recent findings have focused on the relevance of DUBs to cancer development, and pharmacological intervention on these enzymes has become a promising strategy for cancer therapy. In particular, several DUBs are physically and/or functionally associated with the proteasome and are attractive targets for the development of novel anticancer drugs. The successful clinical application of cisplatin in cancer treatment has prompted researchers to develop various metal-based anticancer agents with new properties. Recently, we have reported that several metal-based drugs, such as the antirheumatic gold agent auranofin (AF), the antifouling paint biocides copper pyrithione (CuPT) and zinc pyrithione (ZnPT), and also our two synthesized complexes platinum pyrithione (PtPT) and nickel pyrithione (NiPT), can target the proteasomal DUBs UCHL5 and USP14. In this review, we summarize the recently reported small molecule inhibitors of proteasomal DUBs, with a focus on discussion of the unique nature of metal-based proteasomal DUB inhibitors and their anticancer activity.

## Introduction

**Fig. 1:**
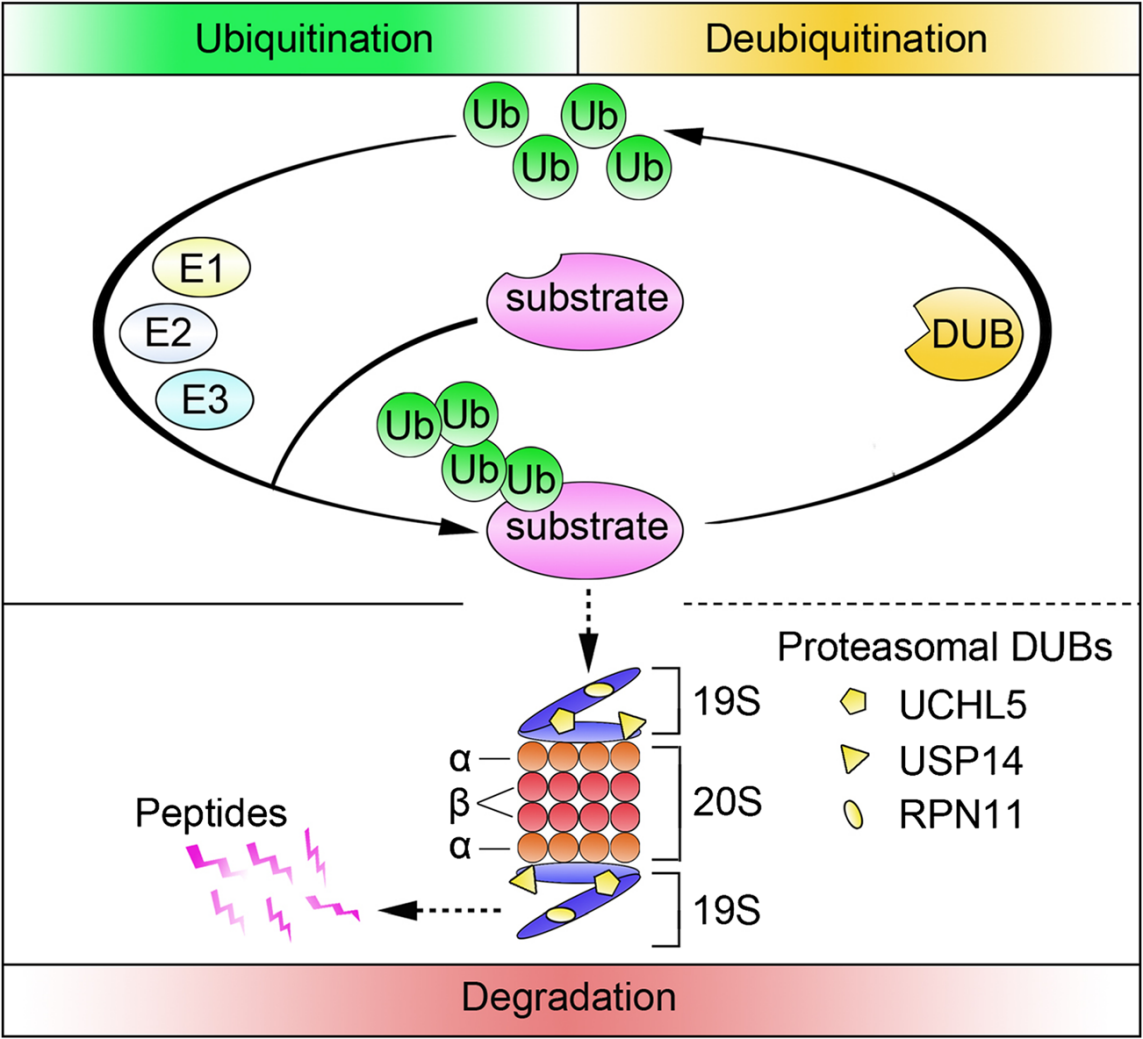
Ubiquitination, deubiquitination, and degradation in the UPS. Ubiquitination is a process substrate tagged by ubiquitin, which is catalyzed by ubiquitin-activating enzyme (E1), ubiquitin-conjugating enzymes (E2), and ubiquitin ligases (E3). Deubiquitination is the reverse process of ubiquitination accomplished through DUBs that remove polyubiquitin chains from target proteins. Polyubiquitinated substrates are recognized, unfolded, and deubiquitinated by the 19S proteasome. Three 19S proteasome-associated DUBs are shown. Degradation of substrates finally occurs within the inner chamber of the 20S proteasome, generally resulting in ~ 7–9 amino acid peptides

The ubiquitin proteasome system (UPS) is the major cellular protein degradation pathway that controls diverse cellular functions, including protein quality control, cell cycle regulation, DNA repair, and immune response [1]. The process of this pathway is divided into ubiquitination, deubiquitination, and degradation of substrates (Fig. 1). Ubiquitination, the attachment of ubiquitin to substrates, is a post-translational modification that mainly regulates the destruction of intracellular protein. In general, this process involves three classes of enzymes: ubiquitin activating enzyme (E1), ubiquitin conjugating enzyme (E2), and ubiquitin ligase (E3) [2]. E1 uses energy from ATP hydrolysis to form a thiol ester bond between its active cysteine and ubiquitin C-terminus. Activated ubiquitin is then transferred to an active-site cysteine residue in the E2 to form the ubiquitin thioester in a similar fashion. Finally, substrate-specific E3 binds ubiquitin-charged E2 as well as substrate and facilitates the formation of an isopeptide bond between the ubiquitin C-terminus and lysine residue on the substrate. In most cases, this ATP-dependent process is repeated to generate a polyubiquitin chain [1, 3]. The degradation of ubiquitinated substrates occurs at the 26S proteasome. The 26S proteasome is a large protein complex composed of a central proteolytic core 20S proteasome and regulatory cap 19S proteasome at one or both ends. The 20S proteasome is a degradation unit with a barrel-like structure that consists of two outer rings formed by the α-subunits and two inner rings formed by the β-subunits. The proteolytic activity is situated in the β1-, β2-, and β5-subunits, conferring caspase-like (C-like), trypsin-like (T-like), and chymotrypsin-like (CT-like) activities, respectively [4]. The conjugation of ubiquitin to substrates is a reversible process, as deubiquitination is catalyzed by deubiquitinases (DUBs) that are capable of removing ubiquitin from protein. This process also facilitates the recycling of free ubiquitin for further use by the UPS.

Protein homeostasis mainly refers to the balance between protein synthesis and degradation, which influences multiple pathways that are essential to the growth and survival of many cancer cells. The high rate of protein synthesis and uncontrolled cell cycle is the hallmark of cancer, making it more reliant on the protein turnover executed by the UPS [5, 6]. Since clinical use of the 20S proteasome inhibitor bortezomib is an effective anticancer agent in multiple myeloma (MM), other components of the UPS, such as DUBs, have proven to be important targets for the treatment of cancer [7]. Recently, it has been shown that potent anticancer properties of some small molecules are due to inhibition of the activities of proteasomal DUBs, suggesting that proteasomal DUBs are new anticancer drug targets [8]. Metal complexes such as copper and gold are known to be able to inhibit the function of UPS *via*, for example but not limited to, targeting the proteolytic sites of the 20S proteasome [9, 10]. More recently, we and others have found that several metal-based drugs can inhibit proteasomal DUBs, including UCHL5 and USP14 [11, 12]. In this review, we summarize the reported proteasomal DUB inhibitors, especially metal-based agents, in the treatment of cancer.

## DUBs and their inhibitors

The human genome encodes nearly 100 DUBs that belong to six different families [13]. Five families are cysteine proteases: ubiquitin C-terminal hydrolases (UCHs), ubiquitin specific proteases (USPs), ovarian tumor proteases (OTUs), Machado-Josephin domain proteases (MJDs), and monocyte chemotactic protein-induced proteins (MCPIPs). The sixth family is JAB1/MPN/Mov34 metalloenzyme (JAMM), which is a kind of zinc-dependent metalloprotease.

DUBs have a profound impact on the stabilization, localization, and activities of proteins involved in many signaling pathways, particularly those frequently altered in cancer, such as DNA repair, cell-cycle control, chromatin remodeling, receptor signaling, and apoptosis [13, 14]. Many studies have highlighted the therapeutic applications of DUB inhibitors. Some irreversible DUB inhibitors, such as ubiquitin vinyl sulfone (UbVS) and ubiquitin aldehyde (Ubal), have been used as research tools for structural studies or activities to detect certain DUBs [15, 16]. However, high molecular weight and limited specificity could be major barriers to using them as therapeutic agents [17]. O-acyl oxime isatins were the first reported small molecule inhibitors of UCHL1 activity and promoted the proliferation of UCH-L1-expressing lung cancer and neuroblastoma cells, suggesting that the effect of UCHL1 is antiproliferative in these cells [18]. In addition, some cyclopentenone prostaglandins, such as delta12-PGJ2 and 15d-PGJ2, inhibit the activities of UCH-L1 and induce ubiquitinated protein aggregation in neuronal cells, which may provide a molecular mechanism linking inflammation with neurodegeneration [19, 20]. Regarding inhibitors of USPs, GW7647 and pimozide non-competitively inhibited USP1 with inhibition constants (Ki) of 0.7 and 0.5 μM, respectively; inhibiting cellular USP1 activity by these agents sensitized human cisplatin-resistant non-small cell lung cancer cells to cisplatin [21]. Another USP1 selective inhibitor (C527) promotes the degradation of ID1 and induces cytotoxicity in leukemic cells [22]. Moreover, some USP7 selective inhibitors, including HBX41,108, HBX19,818, HBX28,258, P022077, P5091, and P22077, have been characterized using fluorescence-based high-throughput screening [23–26]. The USP7 pharmaceutical inhibitors prevent this DUB from deubiquitinating MDM2, resulting in stabilization of p53 in cancer cells [26, 27]. Finally, several analogs of 9-Oxo-9H-indeno [1,2-b]pyrazine-2,3-dicarbonitrile are small-molecule inhibitors of USP8 and induce apoptosis in various cancer cell lines [28].

## Proteasomal DUBs as drug targets for cancer therapy

Three DUBs are associated with the 19S proteasome: Rpn11/POH1, UCHL5/UCH37, and USP14/Ubp6. Rpn11, an integral proteasomal subunit, is a zinc-dependent metalloprotease which belongs to the JAMM family. UCHL5 and USP14 are members of the cysteine protease UCH and USP families, respectively [8]. Both UCHL5 and USP14 are physically associated with the base components of the 19S proteasome; USP14 can bind to the scaffolding protein Rpn1, while UCHL5 is found to bind to both ubiquitin receptors Rpn10 and Rpn13 [29–31]. The activities of proteasomal DUBs are activated upon their assembly into or association with the proteasome through an unclear mechanism. It has been suggested that Rpn11 removes the entire ubiquitin chain at the base from the substrate in a process coupled to degradation [32, 33]. In contrast, UCHL5 and USP14 trim the poly-ubiquitin chain from the distal end [34]. UCHL5 and USP14 are widely believed to have a quality control function preventing the degradation of substrates containing short or non-degradable ubiquitin chains by the proteasome [35]. In cancer cells, RNA interference (RNAi) of Rpn11 decreased proteasome activity and inhibited cell growth by disrupting the assembly of 26S proteasome. In contrast, RNAi of either UCHL5 or USP14 alone did not affect cell growth, proteasome structure, or proteolytic capacity, but increased the rate of protein degradation. Interestingly, RNAi of both UCHL5 and USP14 resulted in inhibition of protein degradation [36].

Rpn11 is essential for cell viability and 26S proteasome’s function [33, 37]. Rpn11 plays an important role in the mitochondrial biogenesis of yeast cells [38]. Overexpression of Rpn11 in mammalian cells exhibits a slower proliferation rate and resistance to cytotoxic drugs [39]. Rpn11 contributes to the regulation of the stability and sublocalization of c-Jun and accordingly affects AP1-mediated gene expression [40]. Preclinical studies show that Rpn11 is highly expressed in MM cells and is inversely correlated with overall patient survival. This report also suggests that Rpn11 inhibitors may improve patient outcomes in MM and overcome 20S proteasome inhibitor resistance [41].

UCHL5 can deubiquitinate and stabilize Smads as well as TGF-β receptor 1 (TGF-β R1) and therefore activate TGF-β signaling [42]. High glucose stimulates PI3K-dependent UCHL5 protein expression, which is required for high glucose-induced deubiquitination and stabilization of TGF-β R1 protein expression in mesangial cells [43]. UCHL5 was also found to deubiquitinate PRP19, an essential RNA splicing factor, and thus could promote cell migration and invasion in hepatocellular carcinoma cell lines [44]. The regulatory function of UCHL5 in apoptosis is associated with alterations in the Bax/Bcl-2 ratio and enzymatic activities of caspase-3/9 [45].

Recent cell culture studies have shown that Usp14 can also stabilize the expression of neurological disease-associated proteins such as Tau and ataxin-3 [46]. However, in USP14-deficient mice, loss of Usp14 did not alter overall neuronal levels of Tau and ataxin-3, but resulted in increased levels of phosphorylated Tau, accompanied by activated phospho-Akt, phosphorylated MAPKs, and inactivated phospho-GSK3b [47]. Several studies suggest that USP14 is a tumor-promoting factor *via* enhancement of the Wnt/β-catenin signaling pathway [48, 49]. USP14 targets the NF-кB pathway by modulating I-кB ubiquitination to promote its degradation [50]. Phosphorylation and activation of USP14 mediated by Akt may provide a mechanism for promoting tumorigenesis in cancer cells with PTEN loss [51]. Moreover, studies have shown high USP14 expression in several kinds of tumors; USP14 exerts a widespread influence on cell proliferation, apoptosis, and tumor metastasis, indicating it as a novel therapeutic target in cancer [52–56].

Due to their involvement and functions in the regulation of important signaling pathways in tumor cells, proteasomal DUBs are emerging as attractive anticancer targets, prompting researchers to discover and develop new, novel proteasomal DUB inhibitors.

### Compounds containing α-β-unsaturated ketone

**Fig. 2:**
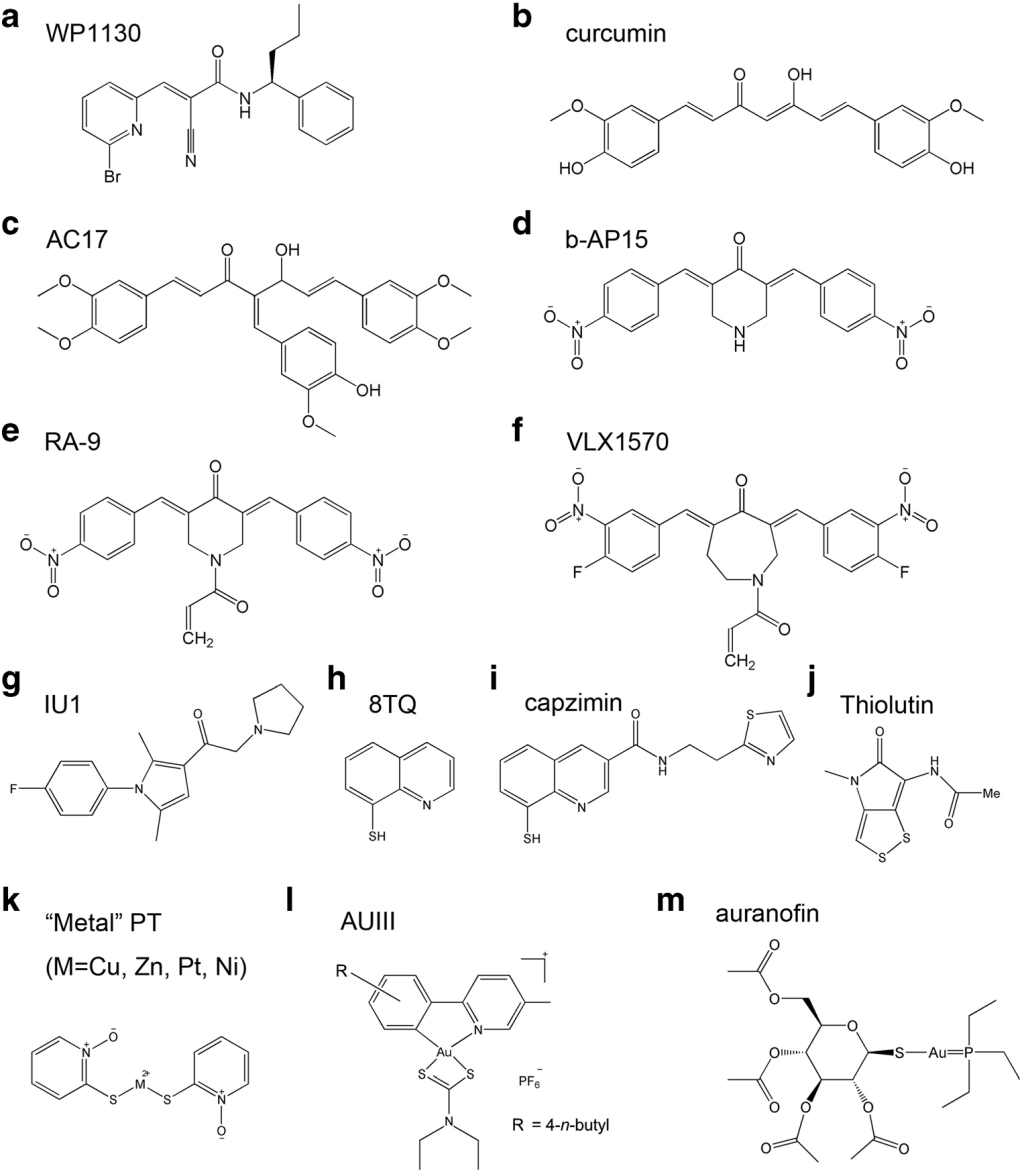
Chemical structures of known small molecule-inhibitors of proteasomal DUBs

The degree of reactivity that occurs in electrophile-nucleophile interactions is based on the “hard and soft acid and bases” principle [57]. It is well-known that α,β-unsaturated ketones can be viewed as relatively soft electrophiles and are believed to react with a subset of soft nucleophilic cysteine thiolates in proteins [14, 58]. Based on this concept, compounds containing α,β-unsaturated ketones (Fig. 2a–f), such as WP1130, curcumin/AC17, and b-AP15/RA-9/VLX1570, have an inhibitory effect on some cysteine DUBs.

WP1130 (Degrasyn) is a small molecule compound that inhibits several cysteine DUBs, including UCHL5, USP14, USP5, and USP9x [59]. WP1130 induced ubiquitination and proteasomal degradation of the anti-apoptotic protein Mcl-1, expected from the direct inhibition of USP9x [60]. The effect of WP1130 on signaling modules in cancer cells was also investigated. Ubiquitination has been reported to regulate protein solubility and accumulation into detergent insoluble complexes within the cell. WP1130 prevents deubiquitination of a subset of kinases, such as Bcr-Abl in chronic myelogenous leukemia (CML), Janus-activated kinase 2 (Jak2), and autophagy-initiating kinase ULK1, resulting in their translocation to the aggresome, thus, failing to participate in signal transduction [61–63]. WP1130 was also shown to efficiently sensitize cancer cells to chemotherapeutic drugs such as bortezomib, cisplatin, and doxorubicin both *in vitro* and *in vivo* [64–66].

Curcumin, a polyphenol derived from the golden spice turmeric (*Curcuma longa*), possesses diverse pharmacologic effects, including anti-inflammatory, antiviral, and anticancer activities [67]. Curcumin has been demonstrated to induce apoptosis mediated by the impairment of UPS, causing accumulation of ubiquitinated proteins in cancer cells [68, 69]. The carbonyl carbons of curcumin can interact with the threonine residue of the β5 subunit, which is responsible for the inhibition of 20S proteasome activity [70]. Curcumin can also directly inhibit ubiquitin isopeptidases, a family of DUBs that is mediated through α,β-unsaturated ketone and two sterically accessible β-carbons [71]. The 4-arylidene curcumin analog AC17 shows improved oral bioavailability, metabolic stability, and moderately potent anticancer activities compared with curcumin. AC17 has been reported to inhibit the activities of 19S proteasomal DUBs, presumably UCHL5 and USP14, but does not inhibit total DUB activities in cell lysates. Moreover, AC17 suppresses tumor growth in a human lung cancer A549 xenograft model, associated with proteasome inhibition, NF-kB blockage, and p53 reactivation [72].

b-AP15 is a specific UCHL5 and USP14 inhibitor identified from a screening of small molecules that induce the lysosomal apoptosis pathway. b-AP15-induced apoptosis was insensitive to p53 status or overexpression of Bcl2 and inhibited tumor progression in both non-solid and solid tumor models, suggesting that its anticancer activity is distinct from that of bortezomib [8]. In addition, b-AP15 was found to trigger apoptosis as well as the unfolded protein response in MM cells and overcome bortezomib resistance [73]. b-AP15 also induced strong oxidative stress by irreversible inhibition of thioredoxin reductase (TrxR); however, TrxR inhibition was not essential for the cell death induced by b-AP15 [74]. RA-9, a chalcone derivative with a structure similar to b-AP15, was reported to inhibit proteasomal DUBs [75] as well as UCHL1, UCHL3, USP2, USP5, and USP8 [76]. Moreover, RA-9 induced apoptosis in ovarian cancer cells and primary cells from donors caused by induction of endoplasmic reticulum (ER) stress, similar to what has been reported for b-AP15 [77]. VLX1570, another chalcone derivative of b-AP15, is a competitive inhibitor of proteasomal DUBs (preferring USP14 over UCHL5) and is currently being studied in a clinical trial for relapsed MM. Changing the central ring structure from a piperidine (six-membered, b-AP15) to an azepane (seven-membered, VLX1570) ring resulted in increased biological activity and inhibition specificity [78]. Indeed, VLX1570 preferentially targets USP14 and exhibits selective cytotoxicity in MM cells, which have a high expression of USP14 [79].

### USP14 inhibitor IU1

After a chemical library screening of 63,052 compounds, IU1 (Fig. 2g) was found to be a specific inhibitor of USP14 that is able to enhance proteasome activity [46]. IU1 did not noticeably induce apoptosis and had a beneficial effect on cell viability correlating with increased clearance of oxidatively damaged proteins [80]. In murine embryonic fibroblasts, IU1 treatment led to an increase in the degradation of the proteasome substrates Tau, TDP-43, and Atx3 that are critical in neurodegenerative diseases [46]. In neurons, IU1 surprisingly triggered calpain-mediated Tau cleavage, but failed to enhance proteasomal degradation of Tau [81]. Additionally, IU1 treatment-attenuated ischemic stroke-induced neuronal injury is associated with enhanced proteasome activity and reduced protein aggregates [82]. Our recent study showing that IU1 significantly decreased the growth of androgen-responsive prostate cancer cells through induction of ubiquitination and degradation of androgen receptor highlights the therapeutic role of USP14 inhibitors [52].

### Rpn11 inhibitors

Quinoline-8-thiol (8TQ; Fig. 2h) is an uncompetitive inhibitor of the proteasomal DUB Rpn11. 8TQ inhibited Rpn11 activity by binding to the catalytic Zn^2+^ ion, which is the active site located within Rpn11’s conserved JAMM domain [83]. However, 8TQ did not distinguish Rpn11 from other JAMMs, such as BRCC36 and AMSH. To improve the potency of 8TQ, structure-activity relationships were explored. A derivative of 8TQ, capzimin (in which “cap” stands for 19S cap, “zim” stands for zinc metalloenzyme, and ‘in’ stands for inhibitor; Fig. 2i), showed a greater than five-fold selectivity for Rpn11 over several other JAMM enzymes. Similarly, the reduced form of thiolutin (Fig. 2j), a disulfide-containing antibiotic, is a zinc chelator that inhibits JAMM metalloproteases such as Rpn11, Csn5, and AMSH [84]. Given the successful development of a number of other metalloenzyme inhibitors, these Rpn11 inhibitors may be promising candidate drugs for cancer therapy [83, 85].

## Metal-based proteasomal DUB inhibitors

Discovery of the biological activity of cisplatin represents a landmark achievement in metal-based anticancer drugs [86]. To date, this platinum-containing drug remains one of the most commonly used agents in the treatment of various cancers such as those of the head and neck, lungs, ovaries, breast, and testicles [87]. The anticancer action of cisplatin is associated with its ability to crosslink DNA and subsequently induces DNA damage and apoptosis in cancer cells [88]. However, severe side effects and drug resistance are often associated with platinum-based agents, which may be major limitations of their use [89, 90]. The successful clinical application of platinum-based complexes has prompted researchers to develop new anticancer agents containing different metals and binding to different targets. Recently, we and others have reported that metal (such as copper and gold)-based compounds can induce cytotoxicity in human cancer cells *via* targeting/inhibiting several DUBs, especially the proteasomal DUBs (Table 1).

**Table 1:** Summary of metal-based proteasomal DUB inhibitors in the literature

Agent	Targets	Cell and animal models	Antitumor mechanisms	Ref.
CuPT	UCHL5 USP14 20S(β5)	MCF-7, HepG2, U266, NCI-H929, GFPu-HEK293, primary AML cells, nude mice bearingHepG2/NCI-H929 xenografts	Induces apoptosis, upregulation of p21, p27, Bax, IκB-α	[107]
AUIII	UCHL1 UCHL3UCHL5	MCF-7, MDA-MB-231, HeLa, non-tumorigenic immortalized liver cells	Induces apoptosis, cell-cycle arrest, anti-angiogenic property	[12]
Auranofin	UCHL5 USP14	MCF-7, HepG2, SMMC-7721, KBM5, KBM5-T315I, GFPu-HEK293, primary AML/CML cells, nude mice bearing MCF-7/SMMC-7721/HepG2/KBM5/KBM5-T315I xenografts	Induces ER stress, apoptosis and NF-κB inactivation, upregulation of c-Jun, p21, IκB-α, downregulation of Bcr-Abl, independent of ROS production	[118, 125, 127]
ZnPT	UCHL5 USP14	U266, K562, A549/DDP, A549, HepG2, SMMC-7721, GFPu-HEK293, primary AML cells, nude mice bearing A549 xenografts	Induces apoptosis, upregulation of p21, p27, independent of DNA damage	[133]
PtPT	UCHL5 USP14	U266, K562, A549/DDP, A549, SMMC-7721, LO2, 16HBE, FPu-HEK293, primary AML cells, nude mice bearing A549/K562 xenografts	Induces apoptosis, upregulation of p21, p27, independent of DNA damage	[134]
NiPT	UCHL5 USP14	U266, K562, A549/DDP, A549, SMMC-7721, KBM5, KBM5-T315I, BaF3-p210-WT, BaF3-p210-T315I, LO2, 16HBE, GFPu-HEK293, primary AML/CML cells, nude mice bearing A549/K562/KBM5/KBM5-T315I xenografts	Induces apoptosis, upregulation of p21, p27, downregulation of Bcr-Abl, independent of DNA damage	[136, 137]

### Copper-based proteasome inhibitors

Copper is an essential element for tumor angiogenesis [91, 92]. Tumor tissues have been observed to possess elevated copper compared with normal tissues [93–95]. Along this line, copper chelation therapy with tetrathiomolybdate is well tolerated and has shown anticancer therapeutic potential in animal models [96]. Disulfram (DSF), a clinically used anti-alcoholism drug, is capable of binding to copper to form a DSF-copper complex, which inhibited the chymotryptic activity of 20S proteasome and induced apoptosis in human breast cancer MDA-MB-231 and MCF10DCIS.com cells [97]. Moreover, DSF-copper complex and copper ion, but not DSF, inhibited 20S proteasome activity, showing that copper is responsible for proteasome inhibition. This is consistent with the hypothesis that DSF is able to carry the copper ion into tumor cells and prevent copper from interacting with many non-specific proteins. Similarly, pyrrolidine dithiocarbamate, clioquinol, and 8-hydroxyquinoline are able to bind with copper, resulting in the formation of an active 20S proteasome inhibitor with promising anticancer effects [98–100]. In addition, several synthesized copper compounds can also inhibit the chymotrypsin-like activity of 20S proteasome and induce cytotoxicity in human cancer cells [101–104]. Diethyldithiocarbamate (DDTC), the active metabolite of DSF, when complexed with copper [Cu(DDTC)_2_] could inhibit purified 20S proteasome and intact 26S proteasomal activities in MDA-MB-231 cells. Interestingly, Cu(DDTC)_2_ only inhibited the activity of purified 20S proteasome at a much higher concentration with an IC_50_ value of ~ 50 μM. However, it inhibited > 90% of cellular 26S proteasome activity at 20 μM in cancer cells [9]. Thus, it has been proposed that inhibition of 19S proteasome might be responsible for the effect of the copper complexes, which needs to be further assessed [9, 105].

Like DSF, pyrithione (PT) spontaneously binds with copper to form an apoptosis inducer, and copper pyrithione (CuPT; Fig. 2k) is used as an active ingredient in antifouling paints [106]. We reported that CuPT was a novel class of the proteasomal DUBs UCHL5 and USP14 [107]. We investigated the effect of CuPT on USP14/UCHL5 activity and its relationship to cytotoxicity. We first compared the toxic effect of the ligand PT or copper ion (Cu) alone and combined in cancer cell lines, including MCF-7, U266, NCI-H929, and SMMC-7721. The results showed that PT or Cu alone had much less effect than that of the combination of PT and Cu. In addition, the combination of PT and Cu induced the accumulation of ubiquitinated proteins in MCF-7 and U266 cells and blocked the degradation of GFPu, a surrogate proteasome substrate, demonstrating the formation of a proteasome inhibitor. Mixing PT with Cu is known to involve the formation of the chelating product CuPT in addition to the self-oxidation product of PT, 2,2′-dipyridyldisulfide (PT2) [108]. However, a previous study reported that PT2 is relatively less toxic than CuPT [109], and we found that the PT and H_2_O_2_ combination did not induce accumulation of ubiquitinated proteins, indicating that CuPT, but not PT2 induces UPS malfunction. We observed that CuPT induced cytotoxicity in MCF7, U266, and HepG2 cancer cells (24 h IC_50_ value all < 0.5 μM) and primary leukemia cells from patients (24 h IC_50_~ 57.03 nM). Moreover, similar to the mixture of PT and copper, CuPT induced UPS malfunction in HepG2 cells and GFP protein aggregation in GFPu-HEK293 cells. Interestingly, we found CuPT can only directly inhibit 20S proteasome activities at high doses (with an IC_50_ value > 1 μM), much higher than its cytotoxic doses in cancer cells. However, 0.5 μM CuPT completely inhibited the activity of 19S proteasomal DUBs and cleavage of Ub4 by proteasomal DUBs. Based on the *in silico* model, CuPT has the potential to interact with both cysteine DUBs UCHL5 and USP14. This has been confirmed by an active site labeling experiment in which CuPT was able to compete with UbVS’s binding with both UCHL5 and USP14. Since there is no crystal structure of Rpn11 available and UbVS is an active site covalent labeling reagent for cysteine DUBs (e.g., UCHL5 and USP14), whether CuPT could interact and inhibit human Rpn11 needs to be investigated in the future.

### Gold complexes

Gold complexes are potential agents that could react with thiols or thiol-containing enzymes. Most thiol-containing enzymes such as glutathione reductases, TrxR, and cysteine proteases are commonly overexpressed in cancer cells, thus providing a potential therapeutic target for gold complexes to treat cancer [110]. Accordingly, recent reports have revealed that both gold(I) and gold(III) complexes can potently inhibit DUB activity in cancer cells [11, 12].

A variety of gold(III) dithiocarbamate complexes have been shown to be potent inhibitors of 20S and 26S proteasome. This inhibition of UPS function is thought to be the key factor for the induction of apoptosis by gold(III) complex in cultured cancer cells and nude mice-bearing tumor xenografts [111–113]. Zhang et al. reported that a [AuIII(C^N)(R2NCS2)] + complex containing a DDTC ligand (abbreviated as AUIII; Fig. 2l) potently inhibited the activities of cysteine DUBs such as UCHL1, UCHL3, and UCHL5 [12]. The inhibitory effect of AUIII on these DUBs has been examined using a fluorescent substrate Ub-AMC, as determined by IC_50_ values with UCHL1 (113 nM), UCHL3 (46 nM), and UCHL5 (75 nM). However, AUIII inhibited the 20S proteasome chymotrypsin-like and trypsin-like activities with the much higher IC_50_ values of 1.9 and 4.3 μM, respectively. After incubation of the peptides of UCHL1, UCHL3, or UCHL5 with excess of AUIII, the mixture was analyzed using liquid chromatography coupled with electrospray ionization mass spectrometry (LC-ESI-MS). The formation of corresponding peptide-AUIII adducts indicated interactions between these cysteine DUBs and AUIII. AUIII (0.9 μM) also inhibited cellular DUB activity in breast adenocarcinoma MCF-7 cells, suggesting that AUIII could effectively enter cells to target DUBs. In addition, AUIII-induced alteration of gene expression is highly similar to that of 15d-PGJ2, which has been reported to be an inhibitor of UCHL1 [20]. In conclusion, gold(III) complexes might display a promising inhibitory effect on DUBs and significant anticancer potential.

Auranofin (AF; Fig. 2m), a gold (I)-containing compound, has been clinically used as anti-rheumatoid arthritis drug for many years. Numerous studies have focused on the anticancer activity of AF. As early as in 1979, AF was shown to exert inhibition on DNA synthesis and cell viability in human cervical adenocarcinoma HeLa cells [114]. A potent *in vivo* antitumor activity of AF has also been demonstrated in a variety of human cancer cell types [115]. AF’s main mechanism of action is thought to be inhibition of TrxR and induction of intracellular oxidative stress [116]. AF has been shown to induce lethal oxidative and proteotoxic ER stress-based UPR responses caused by inhibition of TrxR in cultured and primary chronic lymphocytic leukemia (CLL) cells [117]. Because of the prominent antitumor effect of AF, it has recently been investigated in clinical trials for the treatment of CLL. More recently, it is being investigated in clinical trials for the treatment of recurrent epithelial ovarian and fallopian tube cancer.

We reported for the first time that the gold(I) compound AF is a specific inhibitor of the proteasomal DUBs USP14 and UCHL5 [118]. We found that AF inhibited the cell viability of human hepatoma HepG2 and breast cancer MCF-7 cells with IC_50_ values of 0.17 and 0.41 μM for 48 h, respectively. Moreover, AF treatment for 24 h induces apoptosis in a dose-dependent manner in HepG2 and MCF-7 cells, as observed with fluorescence microscopy and flow cytometry. Next, we found that AF triggers a striking accumulation of ubiquitinated proteins and substrate proteins (p21, c-Jun), indicating UPS inhibition by AF. Importantly, ubiquitinated protein accumulation was detected at an earlier time point (3 h) than PARP cleavage, indicating that apoptosis occurs after proteasome inhibition. However, we observed that AF at a dose as high as 10 μM did not inhibit the CT-like, T-like, and C-like activities of the 20S proteasome. We suspect that AF could indirectly inhibit 26S proteasome function through targeting proteasomal DUBs. We found that AF completely inhibits the DUB activity of 26S proteasome, but had a slight effect on cellular DUB activities. This was also confirmed by computational molecular docking, K48-linked polyubiquitin disassembly, and HA-UbVS competitive binding assay; these experiments showed that AF might inhibit the proteasomal cysteine DUBs UCHL5 and USP14. We also used the thiol-containing compound NAC to block its active site and found that AF-mediated UPS inhibition and cell death was completely reversed by NAC. Most previous studies support that AF-induced cell apoptosis *via* inhibiting TrxR and inducing the generation of reactive oxygen species (ROS). Nevertheless, we found that the antioxidant TBHQ could scavenge ROS induced by AF, but could not rescue AF-mediated proteasome inhibition and cell death. Thus, these results demonstrate that the anticancer activity of AF is associated with proteasomal DUB inhibition rather than ROS generation.

We and others have shown that proteasome inhibition could induce apoptosis in both imatinib (IM)-sensitive and -resistant Bcr/Abl-positive CML cells [119–121]. IM is the most commonly used inhibitor of Bcr-Abl tyrosine kinase, which plays a critical role in the pathogenesis of CML [122, 123]. Unfortunately, some mutations, especially T315I mutation, in the BCR-ABL kinase domain prevent the binding of IM, thus resulting in IM resistance [124]. We explored the potential of the proteasomal DUB inhibitor AF, a clinically used drug, in a treatment of IM-resistant CML cells [125]. We have revealed that AF induces apoptosis in both wild-type and T315I mutation Bcr-Abl-positive CML cells, and inhibits the growth of Bcr-Abl-T315I xenografts *in vivo*. We also found that AF downregulated the expression of both Bcr-Abl protein and mRNA. However, the degree of reduction in mRNA is dramatically less than that in protein, suggesting that AF-induced downregulation of Bcr-Abl transcription is likely partially responsible for the decreased Bcr-Abl protein levels. We observed that the pan-caspase inhibitor z-VAD-fmk could partially reverse AF-mediated Bcr-Abl protein decreases, but did not attenuate ubiquitinated protein accumulation. These results suggest that AF-induced caspase activation by proteasomal DUB inhibition is also required for downregulation of Bcr-Abl protein. Similar to our earlier observation [119], we found that AF-mediated caspase activation and Bcr-Abl downregulation were attributed to proteasome inhibition rather than ROS generation [125, 126]. Recently, we showed that the combination of two clinical drugs, AF and DSF, synergistically enhanced the anticancer effect both *in vitro* and *in vivo*. We demonstrated that the synergistic cytotoxicity of the combination of AF and DSF is associated with UPS inhibition, ER stress, and mitochondrial membrane potential loss-triggered caspase activation and apoptosis [127].

### Pyrithione-metal chelates

Pyrithione (PT) has excellent metal chelating properties. Recently, we have used various metals including zinc, nickel, and platinum (Fig. 2k), complexing with the same ligand PT to compare their effects on the UPS. Zinc pyrithione (ZnPT) has been widely used as a booster biocide in antifouling paints and antidandruff shampoos [128–130], and has also been demonstrated as a potential anticancer treatment [131, 132], but the detailed molecular mechanisms remain unclear. We reported that ZnPT, an FDA-approved pharmacological drug, could target/inhibit proteasomal DUBs (USP14 and UCHL5) without inhibiting 20S proteasome activities [133]. We found that ZnPT efficiently induced apoptosis in various cultured cancer cells and primary cancer cells from leukemia patients and significantly suppressed tumor growth in nude mouse xenografts. This clinically used metal-based complex might have potential as a proteasomal DUB inhibitor for cancer therapy. In addition, we synthesized two other metal complexes, platinum pyrithione (PtPT) and nickel pyrithione (NiPT). We found that the platinum-based agent PtPT targets proteasomal DUBs (UCHL5 and USP14) rather than DNA, which is different from cisplatin [134]. Cisplatin is highly reactive towards DNA because there are two non-leaving groups (NH3) and two fast-rate leaving groups (Cl) around the platinum ion [135]. However, PtPT has two medium-rate leaving groups (S and O) in PT, which allows it to react with protein targets even before reaching the nucleus. We observed that PtPT exhibited greater cytotoxicity than cisplatin in multiple cancer cells and was also highly effective in cisplatin-resistant A549 cells. Moreover, PtPT selectively induced cytotoxicity and UPS inhibition in primary leukemia cells, but not in mononuclear cells from healthy volunteers. PtPT also remarkably inhibited tumor growth in nude mice xenografts without showing any adverse effects. Hence, we have discovered a new platinum-based complex, PtPT, which targets proteasomal DUBs and exhibits better anticancer activities and lower toxicity than cisplatin. The other metal-based complex we synthesized is nickel pyrithione (NiPT). This novel nickel complex can also target proteasomal DUBs (UCHL5 and USP14) and induce apoptosis in cancer cells [136]. Importantly, NiPT also induced UPS inhibition and apoptosis in both IM-resistant and IM-sensitive CML cells. Mechanistically, NiPT induced decreases in Bcr-Abl proteins, which were associated with the downregulation of Bcr-Abl transcription and the cleavage of Bcr-Abl protein by activated caspases [137]. To determine the contribution of metal ion or of ligand to the observed pharmacological effects of NiPT, we compared the effects of Ni^2+^, PT, and NiPT on proteasomal DUB activities *in vitro*. We found that PT and NiPT, but not Ni^2+^, potently inhibited DUB activity, suggesting that PT plays the most essential role in NiPT-induced DUB inhibition.

**Fig. 3:**
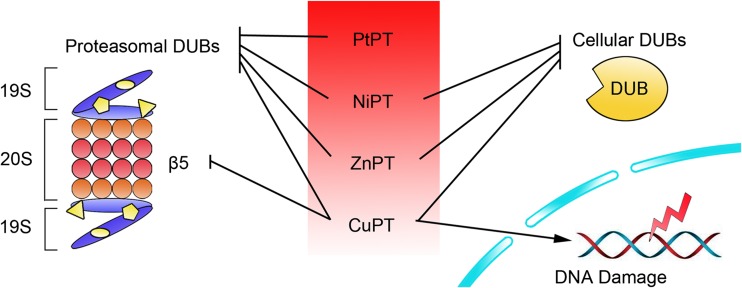
Different selectivities of metal-PT complexes among various enzymes and DNA

Collectively, our data provide compelling support that metal-PT complexes induce typical proteasome inhibition *via* targeting proteasomal DUBs (USP14 and UCHL5), but different metals may have different effects (Fig. 3). CuPT inhibits 20S proteasome activity as well [107], but PtPT, NiPT, and ZnPT do not [133, 134, 136]. Besides targeting proteasomal DUBs, CuPT, NiPT, and ZnPT [107, 133, 136], but not PtPT [134], also inhibit some other non-proteasomal DUBs in the cytoplasm, which is possibly associated with some non-proteasome related cytotoxicity. In addition, PtPT, NiPT, and ZnPT are not DNA damage inducers [133, 134, 136], but CuPT induces DNA damage (unpublished data). Future studies need to be performed to compare the effects of other metal complexes on the activities of 20S proteasome and proteasomal DUBs.

## Conclusions and outlook

Three 20S proteasome inhibitors, bortezomib, carfilzomib, and ixazomib, have been approved by the FDA for treatment of MM, supporting the idea that the components of UPS are attractive targets for anticancer agents. Among susceptible targets in the UPS, DUBs have unique biological functions, tissue-specific expression, and substrate specificities. Thus, DUB inhibitors are expected to be more specific and less toxic. 20S proteasome inhibitors are known to lack efficacy in solid tumors, and their clinical use is hampered by development of resistance and clinical toxicities. Pharmacological inhibition of DUBs has been shown to display powerful antiproliferative and proapoptotic effects in preclinical hematological and solid tumor models and may offer a strategy to overcome bortezomib resistance [26, 73, 83]. Importantly, selective degradation of oncoproteins can be achieved by inhibiting respective DUBs. For instance, wild-type p53-expressing tumors could potentially respond to USP7 inhibitor by affecting MDM2 protein turnover [26]. Inhibition of USP14 may be a highly effective therapy for prostate cancer through degrading AR protein [52]. Based on high throughput screening and structure-based drug design, a number of other DUB inhibitor candidates are expected to be identified. Considering that most DUBs have still not been well characterized, more research is needed on the structural biology of DUBs, identification of their target proteins, and binding modes of their specific inhibitors.

Most DUBs are cysteine proteases, including the proteasomal DUBs UCHL5 and USP14, which could be targeted by metal-based agents. In this review, we have suggested various metal complexes (copper, gold, zinc, etc.) as a new class of potent proteasomal DUB inhibitors. However, the precise mechanism for the specificity of different metal compounds is not yet completely understood. The key to understanding drug specificity is to obtain further chemical analysis of metal-based DUB inhibitors and their binding modes of proteasomal DUBs. Ligands play an important role in modifying the general properties of metal complexes. The exploration of well-chosen ligands endowed with various chemical properties can lead to the improvement of selectivity of metal complexes toward DUBs. Several approaches allow us to move drug development towards more targeted and rational design of metal-based drugs. Metallomic approaches as emerging research areas may contribute to revealing biomolecular binding profiles and the fate of the metal complexes in biological systems [138]. Additionally, proteomic screening and gene expression analysis might lead to a better understanding of the action mechanisms of metal-based anticancer drugs.

Metal complexes present some challenges. For orally administered drugs, adequate absorption and bioavailability must be achieved. Many metal complexes are given intravenously due to their limited solubility in oral formulation and the lack of stability on their passage through the digestive tract [139]. Since excessive bioaccumulation of metals can be highly toxic, the dosage of metal complexes is critical for therapeutic effectiveness and safety. An optimal concentration should be toxic to tumor cells with minimum toxicity to normal cells. However, this is always difficult to achieve with metal complexes [86]. Novel strategies can be developed for the delivery of metal complexes and/or decrease their side effects. Nanoparticles made of polymers (NPs) offers sequential release vectors for antitumor drugs. They allow modulation of drug solubility and distribution, as well as targeting specific cell receptors and reducing side effects of the drug [140, 141]. In addition, an intriguing way to develop metal complex peptide derivatives is based on improved intracellular drug transfer and delivery systems supported by transport proteins. Peptide transporters are integral plasma membrane proteins that seem to be upregulated in some types of tumor. Some gold(III) complex peptidomimetics could preserve the antitumor activities of the gold(III) complex, together with reduced toxic side effects and increased tumor selectivity by targeting the peptide transporters [142].

To our knowledge, thus far, only one proteasomal DUB inhibitor (VLX1570) has successfully entered the clinical trials. This is due to several reasons. Some proteasomal DUB inhibitors (e.g*.*, WP1130) show relatively poor selectivity, targeting not only proteasomal DUBs but also cellular DUBs or other cysteine proteases. Others like USP14 inhibitor (i.e., IU1) are proposed as a therapeutic approach for neurodegenerative diseases or specific tumors by promoting the degradation of neurotoxic or cancer-causing proteins. However, a recent study revealed that it fails to enhance proteasomal degradation of Tau in neurons [81]; therefore, its clinical application is worthy of further testing in animal models. In addition, recently identified Rpn11 inhibitors are attractive anticancer drug candidates that require more preclinical study to evaluate their efficacy and safety. Regarding metal complexes, development of water-soluble metal complexes with potent DUB inhibition effects and metal-based inhibitors of specific DUBs correlating to specific tumor types remain challenging. We expect that novel metal-based complexes could be developed as inhibitors of proteasomal DUBs and be used for cancer therapeutics in the future.
